# Repeatability of cerebral arteriovenous pulse wave propagation in flow-related enhancement MRI

**DOI:** 10.1038/s41598-026-57844-0

**Published:** 2026-07-09

**Authors:** Norman Kornemann, Filip Klimeš, Agilo Luitger Kern, Van Dai Vo Chieu, Jan Wigand Eckstein, Frank Wacker, Julian Glandorf

**Affiliations:** 1 Institute for Diagnostic and Interventional Radiology, Hannover Medical School, Hannover, Germany; 2 Biomedical Research in End-stage and Obstructive Lung Disease Hannover (BREATH), Member of the German Centre for Lung Research (DZL), Hannover, Germany; 3 Department of Radiology, Charité - Universitätsmedizin Berlin, Corporate Member of Freie Universität Berlin, Humboldt-Universität zu Berlin, Berlin Institute of Health, Berlin, Germany

**Keywords:** Flow-related enhancement, Repeatability, bSSFP, Pulse-wave analysis, Health care, Medical research, Neurology, Neuroscience

## Abstract

Flow Related Enhancement (FREE) MRI is a non-contrast technique for temporally resolved cerebral pulse-wave analysis. Clinical implementation requires proven repeatability. We aimed to evaluate the intra- and inter-scan repeatability of FREE-MRI measurements in healthy volunteers. Twenty-four healthy volunteers were scanned on a 3T MRI using a balanced steady-state free precession (bSSFP) sequence. A test-retest protocol was performed: two scans, a break with repositioning, and two more scans. From the resulting pulse-wave delay maps, the arteriovenous delay (AVD) was computed for the anterior (ACA), middle (MCA), and posterior (PCA) cerebral arteries. Repeatability was assessed using Bland-Altman analysis and Spearman correlation. Mean AVDs were 366 ± 51 ms (ACA), 371 ± 55 ms (MCA), and 376 ± 53 ms (PCA). Intra-scan repeatability was variable; the first session (Run 1 vs. 2) showed no significant differences, whereas the second session (Run 3 vs. 4, post-repositioning) showed significant deviation. However, inter-scan repeatability (comparing pooled pre- vs. post-break acquisitions) showed no significant differences after Bonferroni correction. Bland-Altman analysis confirmed that averaging measurements (Before vs. After) narrowed the limits of agreement compared to single-run comparisons, indicating improved stability. FREE-MRI provides quantitative assessments of cerebral pulse-wave dynamics, though single-acquisition precision is sensitive to physiological state. While intra-scan repeatability was high at rest, immediate post-repositioning scans showed significant variability, highlighting the need for a settling period. Crucially, while averaging measurements across sessions (Before vs. After) helps mitigate this noise, inter-scan reproducibility remains limited and protocol-dependent. Despite these limitations, this study provides a foundational step toward establishing a practical protocol for future clinical and longitudinal applications.

## Introduction

MRI of brain perfusion has become increasingly important in the last decade, especially for detection and monitoring of brain diseases such as stroke and dementia^1,2^. Recently, our group introduced Flow Related Enhancement (FREE) MRI, a postprocessing method for cerebral pulse-wave analysis^3,4^. The sequence does not require the use of contrast agents, which may have potential side effects, including gadolinium deposition in the brain^5^. Unlike arterial spin labeling (ASL) techniques such as pseudocontinuous arterial spin labeling (pCASL), FREE-MRI offers assessment of temporally resolved pulse-wave propagation. This might be potentially useful in conditions such as acute ischemic stroke, vascular aging processes, dementia, migraine associated hyperperfusion and brain tumors^6–10^. An assessment of the vascular supply and hemodynamics plays a key role in all of these conditions and influences the subsequent treatment.

The repeatability of magnetic resonance imaging (MRI) measurements is the basis for their use in daily clinical practice. The repeatability of FREE-MRI measurements is currently unknown, impeding the potential introduction of FREE-MRI into a clinical setting. The aim of this study was to evaluate the repeatability of FREE-MRI in healthy study participants.

## Methods

The study was approved by the Institutional Review Board (Ethikkommission der Medizinischen Hochschule Hannover, Nr. 9475_BO_K_2020, Nr. 9651_BO_S_2021). All methods were carried out in accordance with relevant guidelines and regulations, notably the Declaration of Helsinki. Written informed consent was obtained from all study participants prior to imaging.

### MR acquisition

FREE MRI was performed in 24 healthy volunteers (13 males and 11 females, age range: 20–61 years, median age of 28 years). Two axial slices (corpus callosum and 3 cm above) per study participant were acquired using a balanced steady-state free precession (bSSFP) sequence for FREE^3^ on a 3T MR scanner (MAGNETOM Vida, Siemens Healthcare, Erlangen, Germany). For assessment of repeatability, the acquisitions were repeated, resulting in a total of four measurements per slice (two runs before and after a break, during which the participant left the MRI). These slices were strategically positioned at the center of the corpus callosum and 3 cm above it to encompass diverse anatomical structures, including ventricles and basal ganglia with perforating arteries.

The bSSFP sequence was performed using the following parameters: matrix size 128 × 128 (interpolated to 256 × 256 via bicubic interpolation), field of view (FOV) 340 × 340mm^2^, slice thickness 5 mm, pixel bandwidth 1500 Hz/px, TR 3.6ms, TE 1.6ms, flip angle 60°, 500 repetitions, total acquisition time of 3.84 min per slice.

### Postprocessing and calculation of pulse wave delay map

To obtain the pulse wave delay map, the postprocessing described previously has been used and implemented in Matlab (MATLAB 2020b; MathWorks, Natick, Massachusetts, USA)^3,4^. Briefly, a pulse oximeter ensured synchronization by recording the Physiological Monitoring Unit (PMU) signal linked to the imaging readout. The image reconstruction process involved discarding of initial 1280 k-space lines to achieve magnetization steady-state. The recorded PMU signal was used for binning of the acquired k-space data into 15 cardiac phases using a uniform gating window. To mitigate the influence of heart rate variations on temporal alignment, the cardiac cycle was normalized to a standardized duration for each measurement. Averaging of k-space lines was conducted within each phase before image reconstruction. The image reconstruction was performed using an inverse discrete Fourier transform. The resulting whole cardiac cycle comprised 15 temporally-resolved images which depict the pulse wave propagation. Assuming a heart rate of 1 Hz, a temporal resolution of 67ms might be reached. The delay of maximal signal of each signal time series in relation to the maximal signal within a main artery is then calculated in ms, resulting in a pulse wave delay map.

### Image analysis

For the image analysis, a radiologist with 6 years of experience segmented the following 7 regions of interest (ROI) in the averaged image of the whole cardiac cycle, as previously described^3^. Segmentation was restricted to the visible vascular cross-sections of the specific arteries, encompassing the immediate perivascular space, rather than their perfusion territories:

1. Right anterior cerebral artery (ACA R),
2. Left anterior cerebral artery (ACA L),
3. Right middle cerebral artery (MCA R),
4. Left middle cerebral artery (MCA L),
5. Right posterior cerebral artery (PCA R),
6. Left posterior cerebral artery (PCA L),
7. Superior sagittal sinus (SSS).

The arteriovenous delay (AVD) in ms was then calculated for each location by averaging of the delay values inside the defined ROIs in comparison to the superior sagittal sinus.

For the assessment of inter-scan repeatability (Before vs. After Pause), the slices were re-planned on new localizers following subject repositioning. Consequently, ROI segmentation was performed independently for the pre-break and post-break image sets on the non-registered data. However, to eliminate intra-observer spatial variability during the intra-scan assessments, the identical ROI segmentations were applied to the consecutive measurements within each session (i.e., the identical mask was used for the 1st and 2nd measurements, and a separate identical mask for the 3rd and 4th measurements).

### Statistical analysis

To assess the intraprocedural repeatability, a second FREE-MRI run was performed directly after the first measurement. To assess the interprocedural repeatability a third and fourth run were performed after a short break of 10 min while leaving the scanner. Wilcoxon matched-pairs signed-rank test with Bonferroni correction applied for multiple testing (6 comparisons per measurement group), Bland-Altman plots (mean bias and levels of agreement (LoA)) and Spearman correlation coefficient were used to evaluate the repeatability of the AVD at six locations. A P-value < 0.05 was deemed significant for all comparisons other than Wilcoxon matched-pairs signed-rank test. A correlation of > 0.6 was deemed strong^11^. Physiologically implausible negative AVD values and zero values, which may arise from synchronization errors in the cardiac cycle reconstruction, were excluded from the statistical analysis.

## Results

A total of 21 physiologically implausible negative AVD values and zero values out of 576 (3.65%) were excluded. These excluded values were evenly distributed across the major vascular territories (MCA: *n* = 7; ACA: *n* = 7; PCA: *n* = 7) and were clustered within 8 specific subjects. Notably, the majority of exclusions occurred during Run 3 (*n* = 12), immediately following subject repositioning, compared to Run 1 (*n* = 4), Run 2 (*n* = 2), and Run 4 (*n* = 3).

After exclusion of artifacts, the mean AVD across all measurements was 366 ± 51 ms for the anterior cerebral arteries (ACA), 371 ± 55 ms for the middle cerebral arteries (MCA), and 376 ± 53 ms for the posterior cerebral arteries (PCA).

Mean heart rates recorded via the PMU during the respective acquisitions were 65.3 ± 8.0 bpm for Run 1, 66.4 ± 10.7 bpm for Run 2, 70.6 ± 15.7 bpm for Run 3, and 66.6 ± 8.4 bpm for Run 4.

Table 1 summarizes the median AVD with interquartile-range for the right and left anterior, middle and posterior cerebral arteries respectively.

**Table 1:** Median arteriovenous delay (AVD) for the 1st, 2nd, 3rd and 4th FREE measurement evaluated in the right and left anterior, middle and posterior cerebral arteries respectively with the corresponding Wilcoxon P and Spearman r. The 3rd and 4th FREE measurement were performed after a pause during which the patient exited the MRI for a questionnaire.

AVD	Measurement 1, median (IQR), ms	Measurement 2, median (IQR), ms	Wilcoxon 1 vs. 2 *P*-value	Spearman *r* 1 vs. 2, *P*-value	Measurement 3, median (IQR), ms	Measurement 4, median (IQR), ms	Wilcoxon 3 vs. 4 *P*-value	Spearman *r* 3 vs. 4, *P*-value	Wilcoxon before and after pause *P*-value	Spearman *r* before and after pause, *P*-value
ACA R	313 (158–542)	370 (189–517)	0.7540	0.645, 0.0004*	434 (243–703)	345 (224–541)	0.0033*	0.817, < 0.0001*	0.5271	0.698, < 0.0001*
ACA L	304 (169–573)	365 (219–579)	0.8521	0.587, 0.0016*	443 (223–703)	351 (206–550)	0.0029*	0.827, < 0.0001*	0.6487	0.708, < 0.0001*
MCA R	341 (220–565)	359 (158–535)	0.8238	0.824, < 0.0001*	447 (287–732)	352 (241–590)	0.0014*	0.886, < 0.0001*	0.0462	0.759, < 0.0001*
MCA L	331 (208–569)	329 (127–554)	0.7090	0.632, 0.0005*	468 (309–700)	339 (254–543)	0.0003*	0.763, < 0.0001*	0.2800	0.683, 0.0001*
PCA R	335 (220–620)	385 (181–556)	0.5678	0.691, 0.0003*	451 (235–672)	325 (229–533)	0.0007*	0.892, < 0.0001*	0.1058	0.766, < 0.0001*
PCA L	321 (204–561)	396 (201–528)	0.9168	0.649, 0.0004*	448 (235–672)	343 (265–589)	0.0054*	0.883, < 0.0001*	0.1621	0.757, < 0.0001*

*Significant; for Wilcoxon P-values: Bonferroni correction was applied for multiple testing.

Wilcoxon matched-pairs signed-rank test showed no significant differences between the 1st and 2nd measurements. In contrast, comparisons between the 3rd and 4th measurements (performed immediately after subject repositioning) revealed significant differences across all arterial territories, with Run 3 consistently displaying higher AVD values than Run 4 (Table 1).

However, regarding inter-scan repeatability, the comparison of acquisitions Before vs. After the break yielded no significant differences after applying Bonferroni correction for multiple testing (threshold = 0.00833). The lowest observed uncorrected p-value was 0.0462 (MCA R)^12^.

Table 2 summarizes the Bland-Altman analysis for the anterior, middle, and posterior cerebral arteries. The comparison between the 1st and 2nd measurements (1 vs. 2) generally showed a small mean bias close to zero, though with wider limits of agreement (LoA), particularly in the left-sided arteries. In contrast, the comparison between the 3rd and 4th measurements (3 vs. 4) revealed a consistent positive bias across all territories (range: 74.5 to 118.8 ms), indicating that the 3rd measurement values were systematically higher than the 4th. However, the standard deviation of the bias was generally lower in the 3 vs. 4 comparison compared to 1 vs. 2, reflecting less variability between measurements despite the shift. Comparing the averaged values before and after the pause (“Before vs. After”) resulted in a moderate positive bias with limits of agreement that were generally narrower than those observed in the single-run comparisons (1 vs. 2), suggesting that averaging measurements improves reproducibility.

**Table 2:** Bland-Altman mean bias (± SD) and 95% limits of agreement comparing sequential measurements (1st vs. 2nd; 3rd vs. 4th) and interprocedural reproducibility (Before vs. After Pause) in the anterior, middle, and posterior cerebral arteries.

Comparison	AVD Bias (Mean Diff), ms	SD of Bias, ms	Lower 95% Limit of Agreement, ms	Upper 95% Limit of Agreement, ms
ACA R				
1 vs. 2	-10.0	148.5	-301.0	281.0
3 vs. 4	74.5	108.6	-138.4	287.3
Before vs. After Pause	26.0	114.7	-198.8	250.8
ACA L				
1 vs. 2	-19.0	239.5	-488.4	450.4
3 vs. 4	75.3	112.5	-145.2	295.9
Before vs. After Pause	11.0	108.7	-202.1	224.1
MCA R				
1 vs. 2	-2.8	108.3	-215.1	209.5
3 vs. 4	82.5	97.4	-108.3	273.4
Before vs. After Pause	46.0	98.4	-146.9	238.9
MCA L				
1 vs. 2	26.5	240.2	-444.3	497.3
3 vs. 4	118.8	129.5	-135.0	372.6
Before vs. After Pause	18.5	136.5	-249.0	286.0
PCA R				
1 vs. 2	-19.8	161.9	-337.1	297.6
3 vs. 4	77.2	86.8	-92.9	247.4
Before vs. After Pause	71.0	114.7	-153.9	295.9
PCA L				
1 vs. 2	-50.9	205.6	-453.8	352.0
3 vs. 4	76.5	93.3	-106.3	259.4
Before vs. After Pause	45.5	117.0	-183.8	274.8

**Figure 1:**
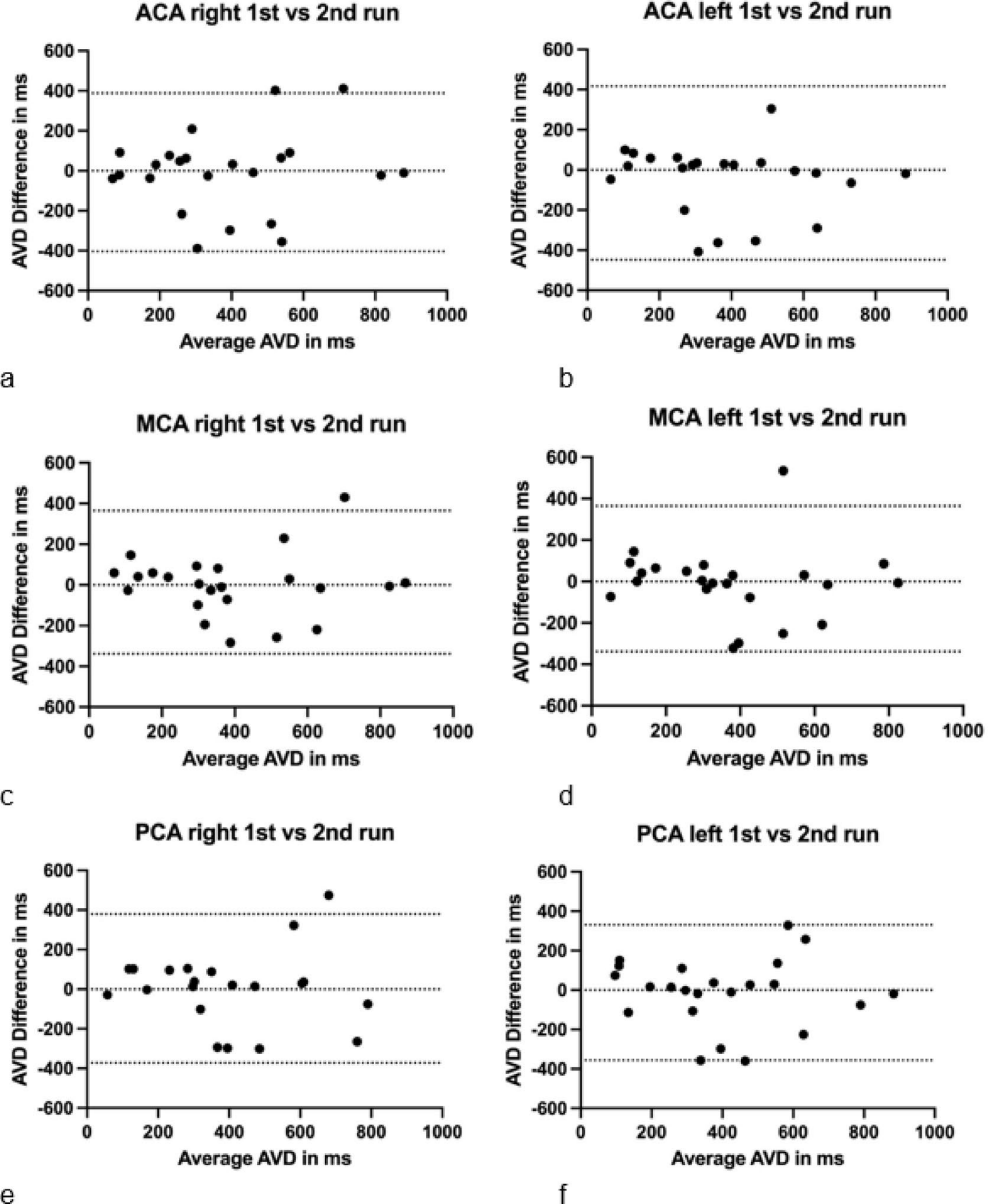
Intraprocedural agreement: Bland-Altman Plots depicting the level of agreement between the 1st and 2nd AVD measurement for the right (A) and left (B) anterior, middle (C, D) and posterior (E, F) cerebral arteries respectively. The middle dotted line represents the mean bias, while the upper and lower dotted lines indicate the 95% limits of agreement.

**Figure 2:**
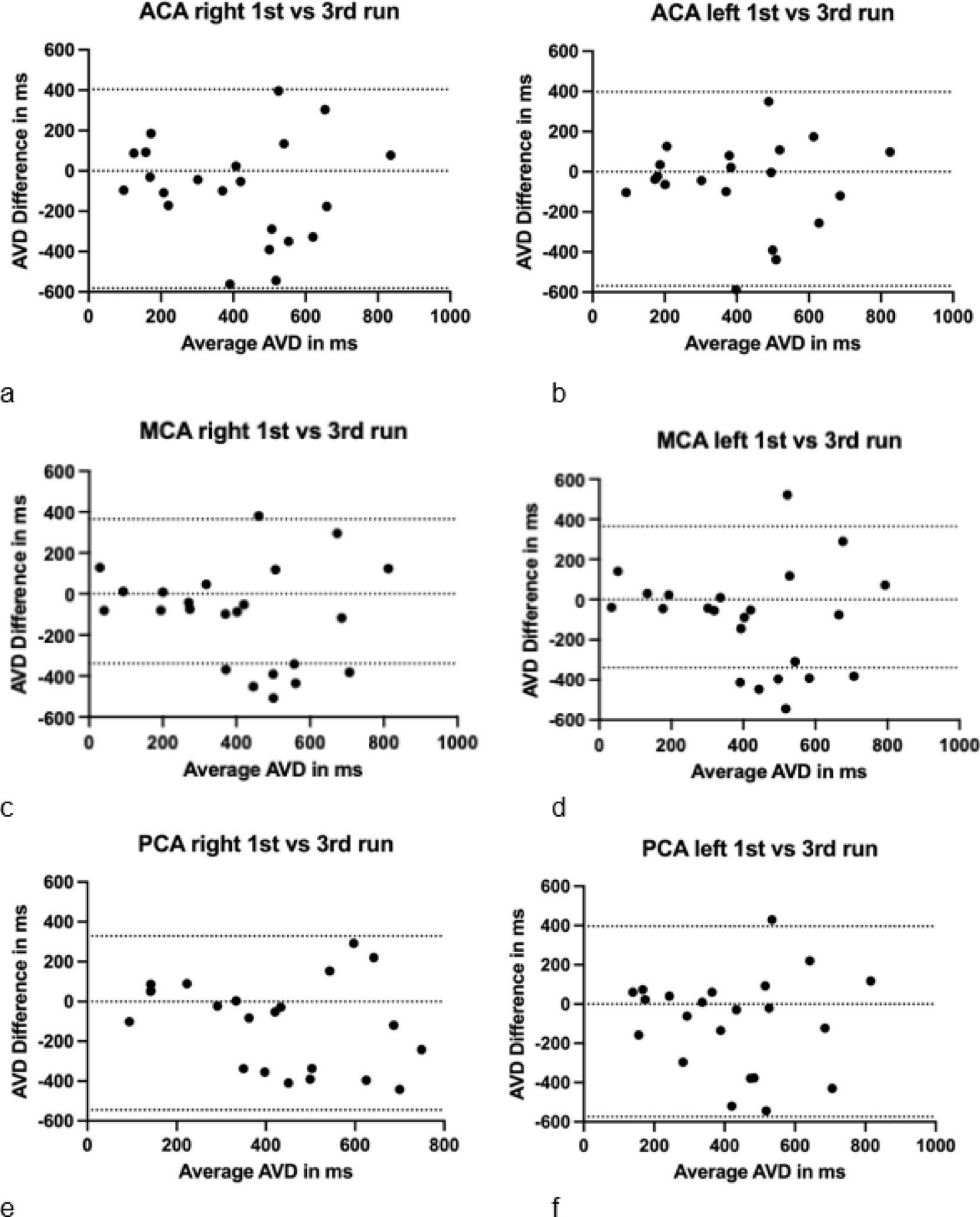
Interprocedural agreement: Bland-Altman Plots depicting the level of agreement between the 1st and 3rd AVD measurement for the right (A) and left (B) anterior, middle (C, D) and posterior (E, F) cerebral arteries respectively. The middle dotted line represents the mean bias, while the upper and lower dotted lines indicate the 95% limits of agreement.

The Bland-Altman plots in Fig. 1 illustrate the intraprocedural agreement and the Bland-Altman plots in Fig. 2 illustrate the interprocedural agreement.

## Discussion

Overall, our study demonstrates variable intra-scan repeatability depending on physiological state. While the measurements obtained from the first two consecutive FREE-MRI scans (resting state) showed no significant mean differences, the scans acquired immediately after repositioning (3rd vs. 4th) exhibited significant deviation, highlighting the sequence’s sensitivity to transient physiological changes. However, crucially for clinical application, the inter-scan reproducibility remained robust. When comparing the sessions before and after the break, no significant differences were found (after Bonferroni correction), and strong interprocedural correlation was maintained. This indicates that despite the patient repositioning and slice replanning, the FREE-MRI method yields reproducible quantitative data, particularly when a settling period is allowed or measurements are averaged.

Our Bland-Altman analysis demonstrates that the precision of FREE-MRI is significantly enhanced by averaging consecutive acquisitions. The wide limits of agreement observed in single-run comparisons (e.g., Run 1 vs. 2) likely reflect physiological noise and the inherent sensitivity of the ‘time-to-peak’ metric. However, the ‘Before vs. After’ comparison, which utilizes the average of two runs per session, showed markedly narrower limits. This suggests that a clinical protocol involving repeated measurements can effectively mitigate physiological variability.

A notable finding in our analysis was the significant difference between the 3rd and 4th measurements, where Run 3 consistently exhibited longer arteriovenous delays than Run 4. This discrepancy, absent in the first session (1 vs. 2), likely reflects a physiological ‘settling’ phenomenon—empirically supported by the elevated mean heart rate and increased variance observed immediately post-repositioning in Run 3. The 3rd run was acquired immediately after the active process of repositioning the subject, which transiently altered vascular tone due to increased sympathetic activity. By the 4th run, the subject had returned to a resting state. It is important to note that the FREE-MRI post-processing algorithm normalizes the cardiac cycle to a standardized duration. This methodological normalization mitigates heart rate variability as a primary source of binning error, suggesting that the prolonged AVDs in Run 3 reflect true physiological shifts in sympathetic vascular tone caused by repositioning. However, alternative explanations must also be considered. The elevation in Run 3 could stem from slice repositioning errors, differences in ROI placement due to independent re-segmentation, or the incomplete achievement of the bSSFP steady state following the 10-minute break. Furthermore, the apparent statistical stability in the ‘Before vs. After’ comparison should be interpreted cautiously; including the potentially biased Run 3 data in the ‘After’ average may artificially mask variances rather than reflect true measurement stability. This confirms that future clinical applications of FREE-MRI should incorporate a mandatory resting period after patient positioning to ensure hemodynamic stability.

To determine the clinical utility of FREE-MRI, the observed precision must be evaluated against expected pathological shifts in cerebral hemodynamics. The limits of agreement widths observed in our averaged inter-scan measurements range from roughly 385 to 535 ms. In conditions involving subtle hemodynamic shifts—such as altered intracranial compliance, where PC-MRI AVD changes by roughly 90 to 100 ms^12^—this measurement error is too wide for reliable single-subject diagnosis. It must also be noted that the absolute AVD measured by FREE-MRI (~ 370 ms) differs fundamentally from pure intraluminal PC-MRI values (~ 89–138 ms); for instance, Bateman et al. reported an AVD of ~ 89 ms, and El Sankari et al. observed an AVD of 18 ± 6% of the cardiac cycle (approximately 138 ms)^13,14^. Even in young healthy cohorts comparable to ours, PC-MRI yields absolute delays of approximately 119 ms^12^. This discrepancy is likely attributable to fundamental differences in the region of interest (ROI) composition and the resulting physiological parameter being measured. While Phase-Contrast techniques typically isolate the intraluminal velocity using strict thresholding, our ROIs encompassed the vascular cross-section and the immediate perivascular environment. Consequently, the measured signal likely incorporates partial volume effects from the surrounding brain parenchyma and cerebrospinal fluid. Therefore, the AVD measured by FREE-MRI does not solely represent rapid intraluminal flow, but rather a composite delay that includes the significantly slower parenchymal pulse wave propagation. This partial volume averaging of intraluminal and perivascular signals naturally exhibits greater latency than pure arterial flow. As supported by recent PC-MRI literature demonstrating short AVDs (calculated median of ~ 119 ms)^12^ even in similarly young cohorts, age and vascular compliance alone cannot account for the prolonged latencies observed in our study. Instead, the four-fold difference between the FREE-MRI composite AVD (~ 370 ms) and pure intraluminal PC-MRI values (~ 89–138 ms) is primarily driven by this inclusion of the slower parenchymal pulse wave.

Additionally, measurement location plays a significant role. While previous studies referenced the cervical Internal Carotid Artery (ICA), our study measured delays starting from the distal intracranial branches (ACA, MCA, PCA) relative to the superior sagittal sinus. These methodological differences in start-point definition likely compound to produce the observed difference in absolute values.

Because FREE-MRI captures this composite delay rather than a pure intraluminal transit time, the specific behavior of this parameter in clinical conditions such as stroke, dementia, and vascular aging remains to be fully elucidated. Future studies must determine how the parenchymal and perivascular components independently alter this composite AVD during disease progression. Nevertheless, in acute ischemic stroke with large vessel occlusion, pathological transit time delays (Tmax) typically exceed 6 s (6,000 ms) in critically hypoperfused tissue^15,16^. While Tmax and FREE-MRI AVD are distinct parameters, the underlying mechanisms are closely connected in large vessel occlusion; an occlusion severe enough to delay microvascular tissue arrival by > 6,000 ms will inherently cause a massive gross prolongation in the macroscopic artery-to-vein transit. Because this expected pathological delta (> 6,000 ms) is much larger than the FREE-MRI measurement error (~ 535 ms), the current averaged precision is theoretically sufficient to detect gross hemodynamic disturbances associated with major cerebrovascular events.

Due to the repositioning of the participant, minimal planning and manual segmentation inaccuracies between the two MR sessions before and after the break were expected.

The stability of the reconstructed cardiac cycle in the FREE algorithm could be potentially enhanced by incorporating parallel imaging and compressed sensing techniques for image reconstruction, which would allow for higher temporal resolution and thereby enhance the precision of AVD measurements. Additionally, sequences using radial or spiral acquisition trajectories may improve k-space sorting and provide greater robustness against participant motion.

The ‘time-to-peak’ metric was used for delay calculation; full waveform analysis (e.g., cross-correlation) may offer superior robustness and will be explored in future work. However, given the discretized cardiac phases (15 phases per cycle), more advanced alignment techniques would currently still be limited by the intrinsic temporal resolution (~ 67 ms per phase).

A limitation of the current study is the small sample size of 24 healthy volunteers. While the results from this population provide valuable information on the reproducibility of the postprocessing method, they may not be generalizable to other populations, such as patients with (brain) diseases. Therefore, potential clinical studies should aim to include larger and more diverse populations to better assess the repeatability and accuracy of the MRI sequence.

In some AVD measurements, negative values were acquired, which might be due to erroneous measurement of the previous cardiac cycle. These were therefore excluded from the statistical analysis. The exclusion of these negative values, however, introduces an asymmetric censoring bias. Because negative values were removed while potentially large positive errors—arising from the same physical mechanisms—remained in the dataset, the reported mean AVD values may be biased upward. The occurrence of these values (3.65%) points to a broader precision limit in the current FREE-MRI implementation. These errors likely stem from PMU timing imprecision, where pulse oximetry may lack the temporal precision required for the exact phase assignment of individual k-space lines. Implementing ECG-based gating in future studies could improve the accuracy of cardiac phase sorting, leading to a more precise reconstruction of the cardiac cycle and reducing both negative and positive temporal errors.

Intra- and inter-observer reliability were not isolated via repeated segmentations of the same images; all segmentations were performed by a single experienced radiologist. However, because identical ROI masks were applied to consecutive intra-scan measurements (Run 1 vs. 2; Run 3 vs. 4), reader variability did not influence the intra-session variance. Conversely, for inter-scan comparisons (Before vs. After), ROIs were drawn independently due to patient repositioning. The reported limits of agreement for the inter-scan comparisons therefore inherently reflect a realistic clinical mixture of technical, physiological, and intra-observer variability. Future clinical validation studies should incorporate multi-observer reliability assessments across different institutions.

## Conclusion

FREE-MRI provides a promising, non-invasive, and contrast-free approach for assessing temporally resolved cerebral pulse-wave dynamics. Our findings demonstrate that single-acquisition metrics are highly sensitive to physiological variability, such as sympathetic ‘settling’ effects following patient positioning. Crucially, however, a protocol incorporating a mandatory resting period and the averaging of consecutive measurements is required to mitigate this noise. Even with averaging, inter-scan reproducibility remains limited and protocol-dependent. While the current limits of agreement remain too wide to reliably detect subtle, individual-level hemodynamic shifts, this study establishes a methodological baseline for FREE-MRI. Because this baseline inherently includes unquantified reader variability, the technique’s current utility is restricted to group-level research, while identifying clear technical targets - such as waveform-based alignment - for its future translation into individual quantitative diagnostics.

## Data Availability

All data was acquired at Hannover Medical School, Germany, and is available in de-identified form from the corresponding author upon request. A formal data sharing agreement is needed. The MATLAB code is available from the corresponding author upon request. A formal code sharing agreement is needed.
